# A randomised trial of nicotine assisted reduction to stop in pharmacies - the redpharm study

**DOI:** 10.1186/1471-2458-12-182

**Published:** 2012-03-12

**Authors:** Taina Taskila, Susan MacAskill, Tim Coleman, Jean-Francois Etter, Mahendra Patel, Sarah Clarke, Rachel Bridson, Paul Aveyard

**Affiliations:** 1 UK Centre for Tobacco Control Studies, Primary Care Clinical Sciences, School of Health and Population Sciences, Primary Care Clinical Sciences building, University of Birmingham, Birmingham B15 2TT, UK; 2 Institute for Social Marketing, University of Stirling and Open University, Stirling FK9 4LA, Scotland, UK; 3 Division of Primary Care, University of Nottingham, D1417, Medical School, Queen's Medical Centre, Nottingham NG7 2UH, UK; 4 University of Geneva, CMU, Institute of Social and Preventive Medicine, 1 rue Michel-Servet, CH-1211, Geneva 4, Switzerland; 5 University of Huddersfield, Queensgate, Huddersfield HD1 3DH, UK

**Keywords:** Smoking, Tobacco Dependence, Controlled Clinical Trials, Randomized, Pharmacists, Harm Reduction

## Abstract

**Background:**

Public policy and clinical treatment in tobacco addiction in the UK has focused on cessation: an abrupt attempt to stop all cigarettes. However, recent evidence suggests that allowing more gradual withdrawal from tobacco or even permanent partial substitution by nicotine replacement therapy (NRT) could lead to net benefits to public health. No jurisdiction has introduced smoking reduction programmes in normal clinical care and the best methods for their implementation is uncertain. Community pharmacists offering smoking cessation services in the UK are ideally placed to implement reduction programmes.

This pilot study aims therefore to examine the feasibility of implementing smoking reduction programme in pharmacies, and also to see if behavioural support and a longer treatment affect the success rate for cessation.

**Design and methods:**

This is a 2 × 2 randomised factorial trial of behavioural support versus no support and short versus standard length reduction programme. The pharmacists will recruit 16 patients per pharmacy, 160 smokers altogether. Pharmacists will randomise each participant by sealed envelopes. In a standard supported programme, the pharmacist will give support for 34 weeks, inviting participants to set a treatment goal and providing advice on how to reduce cigarette use. Participants in the short programme will be given the same advice on how to reduce but will reduce smoking over four weeks. Participants in the no support arms will be given a leaflet that describes the reduction programmes in 4-week and 34-week format. All participants are encouraged to use of NRT to support the reduction. These processes will be measured by recording the number of recruited smokers; percentage of those who reduce and sustain their consumption to at least 50% of baseline value, and the proportion of people who attain 4 weeks abstinence and 6 months abstinence. Interviews will assess smokers' and pharmacists' views on the way the programme ran.

**Discussion:**

This is a pilot study to assess the feasibility of offering smoking reduction programme within pharmacies that offer naturalistic setting to show population benefit from these programmes. Findings from this trial will inform the development of evidence-based treatment for smokers who want to reduce and best approaches to engage reluctant quitters onto the programme.

**Trial Registration:**

Current Controlled Trials ISRCTN 2010-019259-24

## Background

Clinical treatment in tobacco addiction has almost exclusively focused on cessation - an abrupt attempt to stop smoking. Some smokers, however, feel that cutting down is an appropriate way to stop smoking. In the English Smoking Toolkit study, 57% of current smokers reported they were cutting down, of whom 26% were using nicotine replacement therapy (NRT) to assist this [1,2]. The UK Department of Health (DH) new tobacco control strategy proposes supporting smokers who feel unable to quit to reduce smoking as a precursor to quitting. No jurisdiction, however, has introduced reduction programmes in normal clinical care.

The evidence that smoking reduction might benefit public health is derived from clinical trials, surveys, and other observational epidemiological data. A systematic review of RCTs and health economic analysis [2,3] found that smoking reduction programmes doubled long-term abstinence rates, with absolute effectiveness only slightly lower than comparable cessation programmes. The cost per quality adjusted life year was less than £2000 for most age groups, rising to less than £5000/QALY for the oldest.

The US guidance, however, recently concluded there was insufficient evidence to recommend smoking reduction programmes [4]. The main concern is that offering reduction alongside cessation programmes will divert smokers from more effective and cheaper cessation programmes to 'easier' but less effective and more expensive reduction programmes. Second, trials of smoking reduction offered free support and medication in countries where such help for smoking cessation is neither widely available nor free and so trials may have inadvertently enrolled smokers keen on cessation rather than reduction and that would have caused the increase in cessation seen in the intervention groups. Third, the trials took place in specialist clinics and drew in highly motivated participants. Consequently the best methods for implementation of reduction programmes outside of such contexts are uncertain and this need to be testedin naturalistic settings to show population benefit from these programmes.

As recognised in Action on Smoking and Health (ASH) guidance to the NHS [5], specialist stop smoking services in the UK do not have the resources necessary to implement smoking reduction programmes. NHS stop smoking service practitioners treat approximately only 30% of all smokers. If the nicotine assisted smoking reduction programmes were to fulfill their potential, up to 5 million smokers might be helped by them. Specialist stop smoking services, however, would not have the workforce to oversee such large numbers of smokers. Therefore the wider primary care workforce might need to help implement reduction programmes.

Community pharmacists are ideally placed to implement reduction programmes. They already provide smoking cessation services as part of the NHS stop smoking service, treating 16% of smokers helped by the NHS in 2008/9. They recruit patients to the service primarily by providing opportunistic brief advice to smokers and therefore they offer a naturalistic setting to test whether reduction programmes can be implemented alongside cessation programmes.

There are no trials investigating whether behavioural support enhances the success of reduction programmes. There are, however, such trials for cessation programmes, which show that behavioural support increases by 50-100% the efficacy of abrupt cessation programmes [6,7]. Industry-sponsored trials of NRT versus placebo provided regular behavioural support and monitoring lasting 15-30 minutes per visit over nine visits [2,3]. Therefore, it is reasonable to believe that reduction programmes work with support, but it is unclear whether support enhances efficacy or whether it is necessary. One study examined the efficacy of NRT versus placebo in smokers that wanted to quit slowly by reduction [8]. Trial participants received no behavioural support. This trial showed a near trebling of long-term abstinence with NRT relative to placebo (OR = 2.86, 95%CI 1.93 to 2.94). These data suggest support might not be necessary. However, although no behavioural support was offered, this study included three visits to assess reduction and cessation, which might motivate adherence to the programme, and hence this might not be a true study of effectiveness of medication without behavioural support. Determining the optimum level of support and monitoring necessary for efficacy in reduction programmes is a priority for NHS implementation.

One NRT industry sponsored trial enrolled participants who did not intend to stop smoking in the next month and randomised them to reduce over one month then stop or to reduce then stop over the typical 6-9 months adopted in the UK [9]. Despite the lack of intuitive fit between participants' intentions and the short programme, the four week reduction programme was more effective than the nine month programme assessed 12 months from the start. For confirmed prolonged reduction, the Mantel-Haenszel relative risk (RR) (95%CI) was 2.69 (1.08-6.68) and for confirmed prolonged abstinence it was 4.57 (1.00-20.93). Shorter and more manageable reduction programmes may be more effective than the standard six-month programme.

We propose to assess by using mixed method approach whether pharmacists can be trained to implement a nicotine assisted reduction programme, how well they do so, and how this is received by smokers and by pharmacists. We also aim to test whether behavioural support adds to the effectiveness of reduction programmes and whether short or standard length programmes are more effective.

### Aims and objectives

#### Aim

To examine the feasibility of introducing nicotine assisted reduction to stop in pharmacies.

To estimate the efficacy of:

• more rapid versus slower reduction programmes

• behavioural support relative to self-help support only.

### Objectives

#### Primary objectives

1) To examine whether pharmacists can be trained to engage smokers who do not want to quit and enrol them in a smoking reduction programme.

2) To compare the relative efficacy of short versus standard length reduction programmes on smoking reduction and smoking cessation.

3) To examine the relative efficacy of supported versus self-guided reduction programmes on smoking reduction and smoking cessation.

#### Secondary objectives

1) To assess which strategies used in enrolment and implementation of the programmes were successful and unsuccessful.

2) To estimate the proportion of smokers enrolled in reduction programmes who reduce successfully.

3) To estimate the proportion of smokers who are referred to cessation programmes and quit successfully.

4) To obtain trial participants' and pharmacists' views on the value of reduction programmes and how such programmes might be improved in future.

## Methods/Design

### Participant Recruitment

Pharmacists who work for NHS stop smoking services will be recruited from areas of high smoking prevalence in Birmingham and Yorkshire region; those treating at least four new smokers for cessation per month will be eligible. Over 60 pharmacists in Birmingham see this many patients in the NHS stop smoking service and most are recruited by brief interventions across the counter, thus providing a naturalistic setting in which to test the implementation of reduction programmes and integration with other smoking cessation efforts.

We plan to recruit 10 pharmacies and for each to recruit four participants into a reduction programme each month, providing a quota of 16 in four months. We plan two waves of recruitment. Using two waves will allow us to learn lessons from the first wave, which is consistent with the developmental nature of this trial.

Educational outreach visits are effective in changing GP's behaviour [10]. We will make visits to local GPs to inform them about the programme and encourage them to refer reluctant quitters to the reduction programme. GPs will be given referral cards for their local pharmacy provider to give to patients. If the recruitment of participants is particularly slow during the either wave, GP's will be asked to send a letter to their registered current smokers. The letter will inform them that the reduction programme is available at their local pharmacy. It will also give details of how they can take part, should they want to, along with an information leaflet about the study.

Pharmacists will be asked to recruit participants opportunistically as well as receiving referrals from GPs. To assist opportunistic recruitment, pharmacists will display a poster in the window. Pharmacists will be compensated for their time with service support funding.

### Training for professionals

Pharmacists will be trained in two evening sessions; attendance to both training sessions is required. In the first session, presentations will be given on principles of the reduction programmes and practicalities of running the trial from recruitment to the final behavioural support visit. The second training session will give pharmacists the opportunity to practice what was learned in the first session by role plays. The pharmacists will be reimbursed for their time of attending to the training. The research team will keep in regular contact with the pharmacy staff and help deal with problems arising during the trial.

### Inclusion criteria

Participants must meet all of the following inclusion criteria:

1) aged 18 years or older.

2) daily smokers with either a CO of at least 10 parts per million (ppm) at least 15 minutes after last smoking or smoke at least 10 cigarettes or 8 g of loose tobacco as "roll up" cigarettes daily.

3) do not intend to stop in the next month, but are prepared to reduce their consumption with any of the programmes offered.

4) evidence of a personally signed and dated informed consent document indicating that the participant has been informed of all pertinent aspects of the study and consents to participate and be randomised to either arm.

5) have either a telephone or email for follow-up.

### Exclusion criteria

There are no contraindications for smokers using NRT. However, there are situations where caution is required. This trial will be conducted with minimal clinical monitoring using treatment regimens that have not been proven to have a population benefit and therefore we have elected to exclude potential participants who have cautions for NRT and this represents the bulk of exclusion criteria. Participants presenting any of the following exclusion criteria will be excluded:

1. currently using other NRT, bupropion, nortriptyline, mecamylamine, reserpine, or varenicline, or undergoing any treatment for tobacco dependence (e.g. acupuncture) that they are not willing to stop using.

2. unstable angina pectoris, myocardial infarction, acute coronary syndrome, or cerebrovascular accident during the last 3 weeks.

3. severe cardiac arrhythmia

4. currently uncontrolled hyperthyroidism

5. active phaeocromocytoma

6. pregnancy, lactation or intended pregnancy in the coming year

7. a severe acute or chronic medical or psychiatric condition or previously diagnosed clinically important renal or hepatic disease, that may increase the risk associated with study participation or may interfere with the interpretation of study results and, in the judgment of the investigator, would make the potential participant inappropriate for entry into this study.

### Withdrawal criteria

Given the established safety profile of NRT, we do not expect any serious adverse events (SAE) and suspected unexpected serious adverse reactions (SUSARs) due to the medication. Nevertheless, pharmacists are required to report in case of any SAE or SUSARs to Principal Investigator. In the event of an SAE or SUSAR that is judged either possibly, probably, or definitely related to NRT, the prescription for NRT will be withdrawn and not re-instituted in that person (Appendix 1).

### Sample size

This is a pilot trial designed to test the processes, examine implementation issues, and reactions to the programme of those involved. We estimated a sample size of 160 participants recruited in 10 pharmacies would be sufficient to test the feasibility of the study. The outcomes therefore include the percentage of pharmacists that agree to participate, the percentage that are trained, the percentage that actually recruit smokers into reduction programmes. Our most important efficacy outcome (because it is linked with unequivocal health benefits) is smoking cessation. We will record the weekly recruitment rate of patients into the reduction programmes and into NHS stop smoking programmes, comparing the rate of recruitment into stop smoking programmes for pharmacists participating in the trial with those not doing so. This will give evidence on whether smokers are being diverted by reduction. For smokers in each type of programme, we will record the number of contacts made, the time of the pharmacist used in delivering the programmes, the amount of NRT used in each arm, the number of people that try to quit, and the number of people that attend the pharmacist for post-cessation support as a proportion of those that try to quit. We will measure the fidelity to each programme by recording some consultations and analysing their content against the schedule of proposed content. We will record sustained smoking reduction and abstinence, although the trial is not powered to detect worthwhile differences in the abstinence outcomes. Four week and six month abstinence rates will be measured following the Russell standard. We will record the proportion of people that complete the webform/telephone follow up.

Any trial to test the role of behavioural support and shorter versus standard length reduction programmes would need to use six month prolonged smoking abstinence as the outcome [11,12]. A sample of 160 participants is not large enough to provide definitive evidence of efficacy with such an outcome because our systematic review showed that standard length programmes lead to about 7% of participants sustaining six month abstinence [13]. However, we assume that shorter reduction programmes are about twice as effective, as suggested by the Haustein trial. Our most important efficacy outcome here is four week abstinence. Based on our systematic review, we expect about 7% of participants who follow the standard length programme with behavioural support to sustain abstinence for six months, which equates to about 21% achieving 4 weeks of abstinence, our main efficacy outcome. Based on this, we get the following table, which shows that this sample size will have 80% power to detect a rate ratio of 1.7 or 90% power to detect a rate ratio of 1.8 (Table 1), lower than observed in Haustein.

**Table 1:** Efficacy of shorter reduction programmes versus standard length

			80% power	90% power
**Base quit proportion**	**RR**	**Quit proportion in intervention**	**N in each arm**	**N in each arm**

0.21	1.7	0.357	146	195

0.21	1.8	0.378	114	152

0.21	1.9	0.399	91	122

The following are also considerations in the sample size. If we take key process measures like attendance for behavioural support sessions as 63% (observed in the review), with 80 smokers receiving behavioural support, we could estimate this with +/-7% precision with 80% confidence. Asking each pharmacy to treat 16 patients, 4 in each arm, will give a reasonable range of experiences for them and us to evaluate the programmes.

### Allocation to trial arms and treatment

#### Randomisation

Pharmacists will randomise smokers by sealed envelopes. Block randomisation stratified by pharmacy will be used with two blocks of 4 to ensure randomisation to each arm in every pharmacy that recruits at least 4 participants. Although blocks of 4 could become predictable, no pharmacist will recruit sufficient participants to discern the pattern. Telephonists conducting follow up will be blind to treatment allocation but this is an open label trial and participants and therapists will know which arm they are in.

#### Behavioural intervention

**Figure 1:**
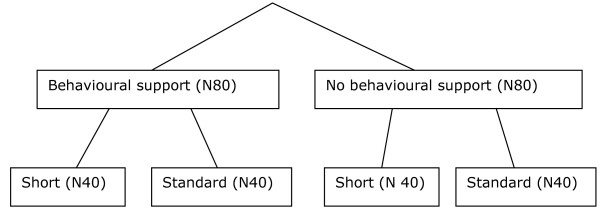
**Randomised factorial design trial (2 × 2) of behavioural support versus no support on smoking reduction**.

This is a 2 × 2 randomised factorial design trial of behavioural support versus no support and short versus standard length reduction programme (Figure 1). It is a pilot study for a later definitive trial.

All participants will receive NRT and be randomised to either behavioural support or no support and either standard length or short reduction; approximately 80 people in each arm.

### Behavioural support arms

#### Supported standard programme

The behavioural support schedule will follow that used in the Nicorette industry trials, providing support and monitoring at baseline, 2, 6, 10, 16, 22, 28, and 34 weeks. The pharmacist giving support in a consulting room will invite participants to set a treatment goal and provide advice on how to reduce cigarettes using unstructured or structured methods of smoking reduction. Unstructured smoking reduction means that participants were not guided as to which cigarettes to eliminate but are left to experiment themselves and without a specific goal except 50% reduction by 6 weeks. Structured reduction means having specific sequential goals for either reducing cigarettes per day or reducing smoking periods. The cigarettes per day methods are either smoke-free periods (SFP) or lengthen the inter-cigarette interval, i.e. the timer method [14,15]. Smoke-free periods concentrates on times when smoking is allowed and when it is not, but the number of cigarettes is not restricted. In the timer method the number of cigarettes per day is restricted and also times when smoking is allowed.

#### Supported short programme

Participants in the 4-week supported programme will be given the same advice and help with reduction but this will be provided on a different schedule. Participants will be seen weekly for four weeks with the aim of reducing smoking to achieve cessation by the end of four weeks. If this fails, reducing to induce cessation at 8 weeks will be the goal. Thereafter, consistent with the pragmatic nature of this trial, participants in the short reduction arm will be allowed to follow the standard reduction regime. Behavioural support will be provided at baseline, weekly to 4 weeks, 6, 8, and 16 weeks i.e. the same number of visits but on a different schedule.

### No support arms

Participants in the no support arms will not be given advice or support by the pharmacist. Instead they will be given a leaflet that describes the reduction programmes (unstructured, cigarettes per day, or smoking periods) and which gives brief advice on using one or more of these approaches, and encourages use of NRT to support this. Participants will be advised to reduce on a schedule that follows the standard programme, if randomised to that arm, or the short programme, if randomised to that arm. The leaflet will advise participants to return to the pharmacy for more NRT to support reduction as needed. This will be dispensed on NHS prescription (following the primary care trust's patient group direction). The outcomes will be monitored without personal visits to avoid the possibility that these are contributing to adherence to the programme.

### Transferring to the cessation programme

The aim of the reduction programme is to help people reduce and pharmacists will be encouraged not to pressure people to stop smoking, while explaining that proven health benefits derive from smoking cessation rather than reduction. However, most people who want to reduce smoking want to do so as a way of stopping [16,17], though rarely with a predetermined timetable. It is for the therapist and the patient to determine whether and when a person is transferred to the cessation programme. Pointers towards possible cessation are:

• Increased confidence that the patient can control her/his smoking.

• Cigarette consumption has fallen to five per day or fewer, or the person is going most of the day without smoking.

Participants in the No-support arms will be asked about their quitting attempt when they return to the pharmacy for more NRT. A key principle of the whole reduction approach is that failed quit attempts do not lead to an end of the programme. If a person moves to cessation and the quit attempts fails, they can resume the reduction programme and continue to control their smoking until they are ready to stop again.

### Length of study

We plan to recruit 10 pharmacies. If they each recruit four participants per month, they will recruit their quota of 16 in 4 months. We plan two waves of recruitment, with final follow up of that cohort one year after the end of recruitment. The second wave of pharmacies will begin recruiting approximately six months after the first wave of recruitment. In total the duration will be 30 months.

The end of the trial is defined as last contact with any trial participant.

### Trial Outcomes

This is a pilot trial designed to test the processes, examine implementation issues, and reactions to the programme of those involved i.e. whether pharmacists can be trained to implement the reduction programme, how well they do so, and how this is received by smokers and by pharmacists. These processes will be measured by:

• The percentage of pharmacists that agree to participate and the percentage that are trained and pass an assessment of competence.

• The monthly recruitment rate of patients into reduction programmes and into NHS stop smoking programmes, comparing the rate of recruitment into stop smoking programmes for pharmacists participating in the trial with those not doing so.

• Recording of some consultations and analysing their content against the schedule of proposed content.

• The proportion of people that would recommend the smoking reduction programme to another smoker and their views on the way that it ran, taken from the 12 month evaluation questionnaire.

We will also examine whether behavioural support is more effective than no support and will investigate whether shorter reduction programmes are more effective than standard length reduction programmes. This will be achieved by measuring self-reported sustained smoking reduction and abstinence. Smoking cessation outcomes will be measured from the time of quitting and we will measure 4 week and 6 month prolonged abstinence following the Russell standard, i.e. by intention to treat and biochemically confirmed [12]. Thus abstinence will be defined as allowing a two week grace period after quit day in which smoking lapses do not count against abstinence and from day 15 onwards, no more than five cigarettes have been smoked. This will be verified by a CO < 10 ppm. Smoking reduction will be assessed by the proportion of people that reduce their consumption to < 50% of baseline value, the mean cigarette consumption at the end of the trial.

As this is a preliminary trial, we will record data to allow us to assess whether the processes of the trial need improvement prior to a definitive trial. We will also examine data on adverse events and drop outs from the programme (Appendix 1).

### Follow up procedures

We propose testing the feasibility of using a follow up method that is unlikely to be perceived by participants as providing them with behavioural support or enhance motivation to adhere. This will be by email/Webform emailed monthly for the 12 months of a participant's involvement in the trial. Monthly follow up will ensure that we can assess whether smokers have started their period of abstinence and schedule verification visits, based on a process outlined for trials of this kind [18]. Web follow up has achieved > 90% follow up in a previous trial [19]. Based on our experience of trials in smokers' clinics, most patients have email addresses and these are useful for follow up. Text reminders will be sent to participants registered for email follow up not responding to the email. For those not using a computer regularly, phone follow up by telephonists will be used so that the pharmacist/therapist is not conducting follow up.

Sustained smoking reduction will be measured as self-reported daily cigarette consumption at 12 month follow up, with a reduction being counted as self-reported consumption lower than at baseline. Sustained reduction will be counted as achieved if during the last four months (i.e. reports at month 9-12) of follow up, a person is smoking less at every follow up occasion than at baseline, measured by self-report. If a monthly report is missing, a person will be counted as having achieved sustained reduction if all other reports show this is the case and the last report is not missing. If two reports are missing, a person will be counted as not having achieved sustained reduction. Mean cigarettes per day at the end of follow up will be calculated from the last follow up only. If cigarettes per day is missing, then this will be replaced by baseline cigarettes per day, but in sensitivity analysis, we will use last observation carried forward.

Participants who have maintained abstinence for 4 weeks will be asked to attend the pharmacy for carbon monoxide (CO) verification if they are in the self-help arm or if they are in the supported arm but are not due for or have ceased to attend visits. Those who have maintained abstinence for 6 months (defined in the same way with a grace period and no more than five cigarettes smoked) will be asked to return for CO verification in the same way. Participants attending the pharmacy for a non-therapeutic reason i.e. to attend for abstinence verification will be paid £20 to compensate them for their time and any travel costs incurred.

### Data analyses

#### Statistics

We will calculate descriptive statistics for the outcome measures comparing these by arm where it is sensible to do so. Risk differences with 95% confidence intervals will be calculated for binary outcomes such as cessation, and for continuous outcomes, such as reduction in cigarettes per day, we will calculate difference in means or difference in change in means. For the cessation and reduction outcomes, we will conduct an intention-to-treat analysis, where all randomized subjects will be included in the denominator. We will also conduct sensitivity analyses with different assumptions for missing data.

The data will be analysed by a multilevel model by examining diversion of smokers from cessation into reduction programmes by comparing uptake of cessation in the pharmacies prior to and during the reduction programmes with pharmacies providing a similar throughput of patients for cessation support but not selected to provide reduction programmes.

Health economic analyses are not required because extensive modelling of different formats of reduction programmes showed that each of these programmes are cost-effective by NHS standards and this was robust to changes in assumptions [3]. However, we will utilise our existing models of the cost-effectiveness of the programme by updating the estimates used in modelling by including these estimates derived from a naturalistic setting. The updated estimates will be used to set the sample size for a definitive trial.

### Qualitative process evaluation

Qualitative research can contribute depth of understanding on why programmes achieve the effects that they do [20,21] and we will use semi-structured interviews with pharmacists and trial participants to determine key factors in relation to initial engagement, ongoing participation and overall response to the programme and the trial. We will purposively selected 4 to 6 pharmacists, some of whom recruited none or few participants and some who recruited many. Semi-structured interviews will investigate factors that influence pharmacist recruitment to the programme and clinical trial, response to training issues, their experiences of implementing the reduction programme and recruiting. The interviews will cover the factors that hindered or facilitated pharmacists' ability to recruit participants and their perceptions of smokers' reactions to behavioural support and medication provided within the programmes. Delivery of the overall programme and behavioural support will be explored including how support delivery is organised within the pharmacy and by whom. Pharmacists will be asked about their understanding of tobacco addiction and its treatment and about tobacco control in general and where smoking reduction fits in, because there is evidence from studies among GPs that these broader attitudes affect the clinical treatments doctors give their patients.

Trial participants will be approached by the research fellow who will select some who have used each of the permutations of support/no support and short/standard reduction programmes. Participants who drop out, reduced and quit smoking, reduced only, or achieved no success with the programmes will be approached for interview. We expect to enrol about 2 people per permutation i.e. about 26 in total. The interviewer will enquire about their motivation for using the programme, their experience of it, and what if any changes occurred to their smoking and why they thought they occurred. Responses to trial follow-up methods will also be explored.

The interviews will be audio-recorded and transcribed with respondents' permission. Interviews will be conducted by the research fellow at a time and location convenient to respondents and trial participants will be offered financial compensation of £20 for their time to acknowledge their contribution to the study. The interviews will be systematically read and interpreted, comparing key features of emergent findings and concepts to minimise bias in interpreting data [22,23]. We will use open coding to identify emergent themes, and categories within these. We will interweave data collection and analysis, conducting later interviews after initial data analysis to provide insights for refining each theme and category.

### Trial Medication

Participants are encouraged to use both a nicotine patch and a short-acting (acute) form of NRT and those who are eligible to pay a prescription charge on the NHS (£7.20) will have to do so for each item dispensed. Combination patch plus acute NRT is becoming a standard dosing regimen for cessation treatment [24], with manufacturers now packaging combined products. This is because of superior efficacy in enhancing cessation of combination over single form NRT [24]. There are fewer trials of NRT in smoking reduction, too few to assess the relative efficacy of the different NRT preparations [2], and no trials of combination NRT versus single form NRT. Therefore the dose regimen is based upon limited trial evidence and extension of the evidence from its use in cessation (reduction to zero cigarettes). A systematic review of placebo controlled short-term trials of nicotine patch and acute NRT show that both reduce daily cigarette consumption in people who are not trying to reduce their smoking and people who are [25]. Both patch and acute NRT appear equally effective in assessing reduction. One long-term trial allowed people free choice between patch, gum, and inhaler and found that gum produced the greater reduction of cigarettes than patch or inhaler [26]. The rationale for patch use is that smokers find regular use of the patch very easy, with typically very high levels of adherence [27], while acute NRT dosing is often sub-optimal [28]. However, acute NRT provides a direct behavioural replacement of cigarettes (such as 'have a piece of gum when you would ordinarily smoke') and this appears important in smoking reduction [29]. This population have relatively low interest in cessation and might need to continue pharmacotherapy for up to a year, so a combination approach seems most likely to produce optimal adherence to regimen and optimal efficacy.

There is no foolproof way to give a person exactly the right dose of nicotine. However, serious problems from too much nicotine replacement are uncommon in smokers. All smokers have had episodes of rapid smoking and can easily identify symptoms resulting from too much nicotine and can cut back. Furthermore, nicotine replacement, particularly in the form of patches, delivers nicotine in a very slow form. Experience of using high dose nicotine patches (up to 63 mg daily) shows that side-effects from 'overdose' are unusual when nicotine is given slowly to smokers [25]. On the other hand, there is good evidence that the smoking reduction without NRT or with an inadequate dose of NRT is much less effective [2]. Bearing these considerations in mind, we propose the following dosing algorithm.

### Initial patch dose

• < 10 cigs/day - 7 mg/24 hour patch or 5 mg/16 hour patch

• 10-19 cigs/day - 14 mg/24 hour patch or 10 mg/16 hour patch

• 20+ cigs/day - 21 mg/24 hour patch or 15 mg/16 hour patch

This dosing guideline should be modified according to exhaled carbon monoxide (CO) reading and patient preference.

• < 10 ppm - 7 mg/24 hour patch or 5 mg/16 hour patch

• 10-19 ppm - 14 mg/24 hour patch or 10 mg/16 hour patch

• ≥20 ppm - 21 mg/24 hour patch or 15 mg/16 hour patch

The patch dose from cigarettes per day will be estimated by exhaled CO. If there is a conflict, the pharmacist should generally use the higher suggested dose. Pharmacists are emphasised that CO levels are typically lower in the morning rising steadily through the day; therefore the dosing guidelines are only approximate

Participants will be advised to remove the patch at night and replace with a new patch each morning i.e. use under 16 hours only, even if the product is a 24 hour patch. One of the most frequent side-effects of patch is vivid dreams or insomnia and using over 16 hours prevents this, with no decrement in efficacy [24]. Reducing adverse reactions is a key aim of treatment that might last for nine months.

### Initial short-acting NRT dose

Participants can have free choice of nicotine inhalator, 2 mg gum, 2 mg sublingual tablets, 2 mg lozenges, or nasal spray.

• For gum, lozenge, or sublingual tablets, participants should replace each missed cigarette with one of their chosen product.

• For nasal spray, participants should take one spray in each nostril to replace each missed cigarette.

• For inhalator, patients should puff as often as is needed. As a rough guide, 40-80 puffs on the inhalator replace one cigarette.

Participants will be strongly encouraged to use both a patch and a short-acting form of NRT concurrently. However, participation in the study depends upon willingness to use NRT in general, so individuals prepared to use only one form of NRT will be allowed to participate. Participants will choose the preferred product together with pharmacists. Participants are allowed to change their NRT at anytime during the trial.

### Dose alteration procedure

Overdose of nicotine is unlikely in clinical practice using the above products. There is still a common perception that it is inadvisable to use NRT and smoke, although the data suggest that this is not true [2,27]. This belief is reinforced by the old labelling that advised people not to smoke while using NRT. The Medicines and Healthcare Regulatory Authority (MRHA) has removed this from all products, but the perception is slower to change, so we will advise pharmacists to reassure patients.

Although nausea or indigestion can be symptoms of overdose, they are common adverse events to oral or nasal NRT and are an unreliable guide to overdose. Likewise, some symptoms such as agitation or restlessness are both symptoms of nicotine withdrawal suggesting under-dosing and of overdose. Pharmacists will therefore be advised to look for definite symptoms of:

• Muscular twitching

• Dizziness

• Confusion

• Rapid pounding heart

• High blood pressure

• Vomiting

• Weakness

Assuming overdose symptoms are not present, then the option for dose alteration will be either to continue with the present patch dose, or to increase the patch dose to the next step i.e. from 7 mg to 14 mg or 14 mg to 21 mg or from 5 mg to 10 mg or 10 mg to 15 mg or 15 mg to 25 mg. Criteria for suggesting an increased dose of patch are as follows

• The patient wants to reduce smoking further

• The patient is finding smoking reduction difficult because s/he is feeling irritable, edgy, and urges to smoke in periods when smoking is 'not allowed' by the programme they are following.

The short-acting forms of NRT have no upper limit of dose for all practical purposes because practical considerations (such as the need to eat), or local irritation from the products, and the sensation of having used too much nicotine within a short time of using the product, naturally limit the dose consumed. The patient will be advised to replace each additional missed cigarette with a short-acting form of NRT as described in the initial dosing. Should a person find that going without cigarettes is difficult; the dose of short-acting NRT should be increased in the following way:

• For gum or lozenge, increase the dose from 2 mg lozenge to 4 mg lozenge.

• For sublingual tablets, participants should use two microtabs at a time.

• For nasal spray, participants should administer two sprays in each nostril to replace each missed cigarette.

• For inhalator, patients should puff as often as is needed.

• Modification of medication regime

Participants who have problems with insomnia or difficulties with vivid dreams will use the patch for 16 hours daily, not 24 hours. Participants who have skin reactions to the patch that are not controlled by switching preparations, emollient and hydrocortisone cream will switch to short-acting NRT only.

• Participants who become pregnant may have their dose adjusted in line with NICE guidance(8) and in accord with the wishes of the participant.

• Participants who show symptoms of overdose will have the dose reduced.

• Participants who give up on their reduction attempt will cease using NRT.

• Participants who experience adverse reactions or adverse events will stop using NRT products, definitively or temporarily, when deemed necessary by the study physician

• Participants who stop the treatment early will not be replaced, and they will be followed-up like the other participants.

Pharmacists are advised to apply same guidelines for patients who come to them with any above issues if they are in the arm of the study which does not give behavioural support.

### Concomitant medication

All medications will be permitted for use concurrently, except those that are proven to help smoking cessation (bupropion, nortriptyline, mecamylamine, reserpine, varenicline), or medications that are unlicensed and for which no interaction data with NRT are available. The NRT itself is aimed at the relief of symptoms of nicotine withdrawal. Should adverse skin reactions occur with the use of the patch, advice will be given on the use of over the counter emollients and 1% hydrocortisone cream, as is standard. Data on all concomitant medication will be recorded.

## Discussion

The findings from the proposed trial are timely and highly relevant in public health as there is a recognized need to support smokers who feel unable to quit to reduce their smoking instead. The Department of Health (DH) proposed a new tobacco control strategy that envisaged 'tailored quit plans' meaning smoking reduction as an intermediate goal without necessarily committing to abstinence. Although the DH has committed to harm reduction, there is no trial showing that allowing reduction alongside cessation leads to greater health benefits than offering abrupt cessation only. This is a pilot study to assess the feasibility of offering smoking reduction programme within pharmacies that offer naturalistic setting to show population benefit from these programmes. In addition, findings from this trial will inform the development of evidence-based treatment for smokers who want to reduce and best approaches to engage reluctant quitters onto the programme.

### Procedures for handling the data

#### Data Management

The trial will run as part of the portfolio of trials in the Primary Care Clinical Research and Trials Unit (PCCRTU), an NIHR recognised trials unit in Primary Care Clinical Sciences at the University of Birmingham. The data management will be run in accord with the standard operating procedures (SOPs), which are fully compliant with the Data Protection Act and International Conference on Harmonisation (ICH) and Good Clinical Practice (GCP). The source documents for the trial will be the case report forms (CRFs) which will be stored in the pharmacies in a locked cabinet in a locked office. The trial database will be securely held and maintained by the PCCRTU. Data cleaning will take place by a series of logical checks on the electronic data. (For example, a person cannot be recorded as prolonged abstinent smoker at 6 months if they were not in such a state at 8 weeks). Discrepant records will be checked with the source documents and the database amended if necessary. On completion of the trial and data checking, the CRFs will be transferred to Modern Records, a secure archiving facility at the University of Birmingham, where they will be held for 15 years and then destroyed. The database will be anonymised and a secure compact disc containing the link between identification number and patient identifiable information will be stored in modern records.

### Data Protection and Confidentiality

Data will be kept in accordance with the Data Protection Act and the trial registered with the Data Protection Act website at the University of Birmingham. The SOPs of the trials unit will be followed, which are designed to protect patient confidentiality. Patient identifiable data will be shared only within the clinical team on a need-to-know basis to provide clinical care and ensure good and appropriate follow up. Patient identifiable data will also be shared with the GP and approved auditors from the Research Ethics committee (REC), NHS Research and Development, or the MHRA will also be able to see patient identifiable information. Otherwise, confidentiality will be maintained and no one outside the trial team will have access to either the CRFs or the database. No data relating to individuals will be identified in publications.

### Ethical approval

The trial will be conducted in compliance with the principles of the Declaration of Helsinki (1996), the principles of ICH-GCP and run in accord with EU Clinical Trials Directive and all of the applicable regulatory requirements. The study protocol and other documentation have been approved by the Birmingham East, North and Solihull Research Ethics Committee, 14^th ^of June 2010 (REC reference number: 10/H1206/22) and amendment on 21 November 2011 (Amendment number: AM01). Any subsequent protocol amendments will be submitted to the REC for approval and the other bodies if necessary. We will comply with ICH-GCP Guidelines over the reporting of adverse events, serious adverse events, and suspected serious adverse reactions (SUSARS). In addition we will provide the REC with progress reports as well as a copy of the Final Study Report.

### Dissemination of the results

The trial results will be written up for submission to peer reviewed scientific journals and presented in national and international conferences. No data relating to individuals will be identified.

## Competing interests

TT has no competing interests. PA has done consultancy work for Pfizer, McNeil, and Xenova/Celtic. TC has done consultancy work for Johnson and Johnson and Pierre Fabre Laboratories, MP has no competing interests. SM has no competing interests, RB has worked with Pfizer on unrelated research projects. JFE has no competing interests. SC has no competing interests.

## Authors' contributions

TT participated in the design of the study and drafted the manuscript. PA conceived of the study, participated in its design and helped to draft the manuscript. TC, JFE, SM, RB, MP, SC and DW all participated in the design of the study and reviewed the manuscript. All authors read and approved the final manuscript.

## Funding

The study is funded by the award of a grant from the Prevention Research Advisory Board of the National Prevention Research Initiative (NPRI)

## Appendix 1 Adverse Event Reporting

### Definitions

#### B.1. Adverse event

An AE is any untoward medical occurrence in a clinical investigation participant administered a product or medical device; the event need not necessarily have a causal relationship with the treatment or usage. Examples of AEs include but are not limited to:

• abnormal test findings,

• clinically significant symptoms and signs,

• changes in physical examination findings,

• hypersensitivity, and

• progression/worsening of underlying disease.

Additionally, they may include the signs or symptoms resulting from:

• drug overdose,

• drug withdrawal,

• drug abuse,

• drug misuse,

• drug interactions,

• drug dependency,

• exposure in utero.

Failure of expected pharmacological action or therapeutic benefit alone (i.e. lack of efficacy) is not necessarily an AE.

#### B.2. Definition of serious adverse event

A serious adverse event or serious adverse drug reaction is any untoward medical occurrence at any dose that: results in death, is life-threatening (immediate risk of death), requires inpatient hospitalisation or prolongation of existing hospitalisation, results in persistent or significant disability/incapacity, and/or results in congenital anomaly/birth defect. An important medical event may not be immediately life-threatening and/or result in death or hospitalisation. However, if it is determined that the event may jeopardize the participant and may require intervention to prevent one of the other outcomes listed in the definition above, the important medical event should be reported as serious. Examples of such events are intensive treatment in an accident and emergency department or at home for bronchospasm; blood dyscrasias or convulsions that do not result in hospitalisation; or development of drug dependency or drug abuse. Serious adverse events will be those that occur during the period of medication use or within 7 or more days of ceasing medication use.

#### B.3. Definition of suspected serious adverse reaction (SSAR)

Means an adverse reaction that is classed as serious and which is consistent with the information about the medicinal product in question set out (in the case of a licensed product, in the summary of product characteristics for that product.)

#### B.4. Definition of suspected unexpected serious adverse reaction (SUSAR)

Means an adverse reaction that is classed as serious and which is not consistent with the information about the medicinal product in question set out (in the case of a licensed product, in the summary of product characteristics for that product)

#### B.5. Monitoring and reporting adverse events

NRT is a well tried and tested medication and there are minor common and well known side-effects that it will not be practical or useful to record. For example, oral NRT almost universally causes burning in the mouth that most people find initially unpleasant but become used to in due time. Where adverse events are listed in SPC as expected and are classified as mild, pharmacists will not be required to record these on the adverse event form.

For all other adverse events (i.e. moderate or severe or possibly serious), the investigator will pursue and obtain information adequate both to determine the outcome of the adverse event and to assess whether it meets the criteria for classification as a serious adverse event requiring immediate notification to the sponsor, the NHS R&D office, and the research ethics committee. The investigator will assess causality. For adverse events follow-up by the investigator is required until the event or its sequela resolve or stabilise.

#### B.6. Severity Assessment

The treating clinician or investigator will use the adjectives mild, moderate, or severe to describe the maximum intensity of the adverse event. For purposes of consistency, these intensity grades are defined as follows:

• Mild- Does not interfere with participant's usual function.

• Moderate- Interferes to some extent with participant's usual function.

• Severe- Interferes significantly with participant's usual function.

Note the distinction between the severity and the seriousness of an adverse event. A severe event is not necessarily a serious event. For example, a headache may be severe (interferes significantly with participant's usual function) but would not be classified as serious unless it met one of the criteria for serious adverse events, listed above.

#### B.7. Exposure In Utero

The license for NRT does not exclude use in pregnancy and NICE guidelines allow such use. We will exclude pregnant or breast feeding women because the dose and format of NRT advised for pregnant women is different from that used in our protocol. Consequently, we will adjust the dose of NRT should a woman become pregnant during treatment. As NRT use in pregnancy is routine in NHS practice, we will not follow up such women to determine the outcome of pregnancy in such cases.

#### B.8. Causality Assessment

The pharmacist's or investigator's assessment of causality must be provided for all adverse events (serious and non-serious). An investigator's causality assessment is the determination of whether there exists a reasonable possibility that the investigational product caused or contributed to an adverse event. If the investigator's final determination of causality is unknown and the investigator does not know whether or not investigational product caused the event, then the event will be handled as "related to investigational product" for reporting purposes. If the investigator's causality assessment is "unknown but not related to investigational product", this should be clearly documented in the CRF. In addition, if the investigator determines a serious adverse event is associated with trial procedures, the investigator must record this causal relationship, as appropriate, and report such an assessment in accordance with the serious adverse event reporting requirements, if applicable.

#### B.9. Evaluation of AEs for causality

• Not Related. Onset of the event as relative to administration of the product, is not reasonable; or, another cause itself can explain the occurrence of the event

• Unlikely to be related. Onset of the event as relative to administration of the product is possible but another cause itself can explain the occurrence of the event or there are no reasonable grounds for suspecting that the product could have caused the event.

• Possibly related. Onset of the event as relative to administration of the product is reasonable; however the event could have been due to another, equally likely, cause

• Probably related. Onset of the event as relative to administration of the product is reasonable and is more likely explained by the drug than by any other cause.

• Definitely related. Onset of the event as relative to administration of the product is reasonable and there is no other cause to explain the event; or a re-challenge (if feasible) is positive.

AE is classified as "not related" or "unlikely to be related" in case the patient has stopped using NRT seven days or longer at occurrence of the event.

The pharmacists/clinicians' responsibilities and processes for evaluating AEs

The participant will be encouraged to report AEs to the pharmacist, who will manage these in the normal manner for reported AEs. The pharmacist completes a form called: "Undiagnosed health problem or hospitalisation/death/disability log" in the CRF. In the case of SUSARs or SAEs, the pharmacist will report to the PI or other member of the investigating team within 24 hours of becoming aware of such a possible occurrence. In case the research team becomes aware of the occurrence first, the Principal Investigator will inform the pharmacist and fill in the log in the online CRF. It is pharmacist's responsibility to ensure that both versions of the CRF log (online and paper version) are accurate.

#### B.10. The CI/PI's responsibilities and processes for evaluating AEs

Each AE reported to the PI will be evaluated for seriousness, causality, expectedness and severity. The responsibility for this will lie with Dr Paul Aveyard, the PI..

Reporting to the sponsor will be required where the AE has a possible causal relationship to the trial intervention, and/or is unexpected. In this case Dr Aveyard will report the event to the sponsor as soon as being made aware of the event. An initial verbal report can be made but will be followed promptly with a detailed written report on the trial SAE form The copy of the form will be filed in the trial master file.

Timeframes in which the Sponsor will submit expedited reports to the Research Ethics Committee (REC) and to the Medicines and Healthcare Products Regulatory Agency (MHRA)

##### B.10.1. *Fatal/life threatening SUSARs*

The sponsor will inform the REC of the above as soon as possible, but no later than 7 calendar days after he has first knowledge of the minimum criteria for expedited reporting.

##### B.10.2. *Non-fatal and non-life threatening SUSARs*

The sponsor will report all other SUSARs and safety issues to OXREC as soon as possible but no later than 15 calendar days after he has first knowledge of the minimum criteria for expedited reporting.

##### B.10.3. *Reporting other safety issues*

A letter entitled Safety Report will be sent to the REC where other safety issues also qualify for expedited reporting by the sponsor. The Co-ordinator of the main REC will acknowledge receipt of safety reports within 30 days.

## Pre-publication history

The pre-publication history for this paper can be accessed here:

http://www.biomedcentral.com/1471-2458/12/182/prepub
